# Coordinated Regulation of Mechanical Behavior and Residual Stress of 9Cr-3W-3Co Steel Based on Jominy Test

**DOI:** 10.3390/ma18112508

**Published:** 2025-05-26

**Authors:** Hongru Lyu, Lin Zhu, Qingxian Ma, Jie Huo, Jiamian Liu, Zhuolin Wang

**Affiliations:** 1 State Key Laboratory of Clean and Efficient Turbomachinery Power Equipment, Department of Mechanical Engineering, Tsinghua University, Beijing 100084, China; lv-hr23@mails.tsinghua.edu.cn (H.L.); liu.jiamian@foxmail.com (J.L.); zl-wang21@mails.tsinghua.edu.cn (Z.W.); 2 China First Heavy Industries, Qiqihar 161042, China; zl508@126.com

**Keywords:** Jominy test, hardenability, residual stress, mechanism analysis

## Abstract

Heat-resistant steel 9Cr-3W-3Co is one of the most important materials of advanced ultra-supercritical units. Investigating the quenching performance of 9Cr-3W-3Co material and optimizing its post-quenching microstructural mechanical properties and residual stress distribution are crucial for ensuring the service performance of large forgings. In this paper, the relevant research was carried out based on the combination of numerical simulation and the Jominy end-quenching test. The microstructure evolution and mechanical properties formation mechanism under different austenitizing temperatures were studied first. Furthermore, considering the residual stress distribution, the heat treatment parameters were optimized. The results showed that the martensite, grain refinement, and carbide distribution of the material were the key factors affecting the hardness after the quenching process. When the austenitizing temperature was 950 °C, a hardness of more than 35 HRC can be obtained within a 50 mm depth after quenching. Meanwhile, on the basis of balancing thermal stress and phase transformation stress, the maximum residual stress decreased by 11.8% compared with that obtained at a 1000 °C austenitizing temperature, dropping to 608 MPa.

## 1. Introduction

To achieve energy-saving and emission-reduction goals, ultra-supercritical (USC) power plants, characterized by a large capacity and high operational parameters, enhance thermal efficiency by optimizing boiler steam temperature and pressure levels. The high-pressure rotor forgings in steam turbines, typically weighing between 10 and 60 tons, constitute the largest high-temperature components in USC units. Under extreme conditions, including high rotational speeds, significant cyclic loading, furnace-generated elevated temperatures, and associated thermal stresses, the forgings must serve at high speeds for more than 20 years reliably [1]. The particularity of the above dimensions and operating conditions put forward requirements for exceptional performance in critical properties of the material such as high-temperature creep resistance, low-cycle fatigue strength, and fracture toughness [2]. The superior microstructural stability of 9–12% Cr steels confers excellent high-temperature creep strength [3]. Nevertheless, conventional materials including T/P92, E911, and P122 are incapable of meeting the operational demands at 630–650 °C, unless expensive high-temperature alloy production methods are implemented. Consequently, the development of advanced rotor steels has emerged as a critical bottleneck restricting the construction and technological advancement of ultra-supercritical (USC) power units. The 9Cr-3W-3Co martensitic heat-resistant steel represents an improved version of conventional 9Cr steels, where molybdenum (Mo) has been eliminated, while the contents of tungsten (W), cobalt (Co), copper (Cu), and boron (B) have been increased. This advanced material exhibits a service temperature exceeding 630 °C and demonstrates several superior properties compared to other heat-resistant steels, including lower manufacturing costs, a reduced thermal expansion coefficient, excellent high-temperature creep resistance, outstanding oxidation resistance, and enhanced fatigue performance. These combined advantages make it one of the most economically viable and practically attractive materials, garnering significant research attention [4,5,6]. Due to its high alloy content, 9Cr-3W-3Co undergoes a complete microstructural transformation into martensite during post-forming quenching, which is accompanied by significant volumetric expansion. The superposition effect of structural stress, induced by the volumetric changes during phase transformation, and thermal stress, resulting from non-uniform and rapid temperature gradients within the component, generate significant internal quenching stresses. When these stresses exceed the material’s yield strength, quenching cracks would be prone to initiate [7]. Moreover, the release of substantial residual quenching stresses during subsequent machining processes can induce exacerbated surface cracking susceptibility and dimensional distortion, adversely affecting the final quality and precision of components.

Extensive studies have focused on microstructural evolution and mechanical property enhancement during quenching. Zhu H.B. et al. [8] studied the quenching behavior and strengthening mechanisms of ultra-high-strength steel with a 0.25% carbon content. They found that the high density of geometrically necessary dislocations (GNDs) during martensitic transformation plays a central role in transformation strengthening. Enhanced cooling rates promote greater hardness. When the cooling rate exceeds 5 °C/s, both hardness and GND density tend to stabilize; however, excessively high cooling rates may induce plate cracking. Wang B.B. et al. [9] summarized factors affecting hardenability, concluding that increased cooling rates lead to refined martensite lath sizes. Further, the austenitizing temperature critically governs hardenability through dual mechanisms, (a) promoting complete carbide dissolution into the austenitic matrix, and (b) inducing grain growth while achieving improved elemental distribution. The mathematical model proposed by Jin M. et al. [10] transformed the prediction problem of the hardenability curve into the problem of solving the hardenability coefficient and established the analytical function expression describing the hardness distribution curve of quenched steel in circular sections. Zhu Y.F. et al. [11] performed computational simulations of the end-quenching behavior in 18CrNiMo7–6 steel and confirmed the superior predictive capability of the model proposed by Jin M. et al. for medium-low carbon steel hardenability curves with experimental validation. Lu S.M. et al. [12] developed a finite element model of the coupling temperature and microstructure evolution during the end-quenching of SA508Gr.4N steel. Their experimental results revealed a transformation from fully martensitic to martensite/bainite mixed microstructures with increasing distance from the quenched end surface. Yang Z.Y. et al. [13] systematically analyzed the factors contributing to the high hardenability of 18CrNiMo7–6HL steel, including inclusions, chemical composition, grain size, martensitic transformation tendency, and heat treatment parameters. The microstructural analysis revealed that although Cr, Mn, Mo, and Ni alloying elements effectively enhance hardenability through solid solution strengthening and delayed transformation kinetics, differential phase transformation rates induce severe quenching stresses, thereby elevating the crack initiation propensity. Based on the aforementioned phenomenon, it is recommended to implement a pre-quenching cooling process involving 1–3 min of air cooling prior to oil immersion. The pre-cooling treatment can effectively regulate the cooling rate gradient along the depth direction, thereby reducing the detrimental effects of quenching-induced stresses. To mitigate quenching-induced internal stresses, Feng X.W et al. [14] investigated the temperature and stress distributions during the quenching process of H13 steel, identifying that the maximum residual stresses consistently localized at the surface region after quenching, which significantly increases the susceptibility to the in-service failure of the quenched components.

In thermal manufacturing processes, precisely controlling the temperature variation profile to achieve ideal microstructural phase transformations while minimizing excessive residual stresses holds profound implications for ensuring component service performance. Hardenability, defined as the capacity of steel to obtain martensite when quenched under certain austenitizing conditions, is an important criterion to evaluate microstructural evolution efficiency. The end-quench (Jominy) test has been widely adopted for hardenability assessments, providing a scientific reference for the optimization of heat treatment processes. Current research predominantly focuses on individual or dual aspects among quenching residual stress, microstructural evolution, and mechanical properties, with limited studies conducting a comprehensive analysis of all three factors simultaneously.

**Figure 1:**
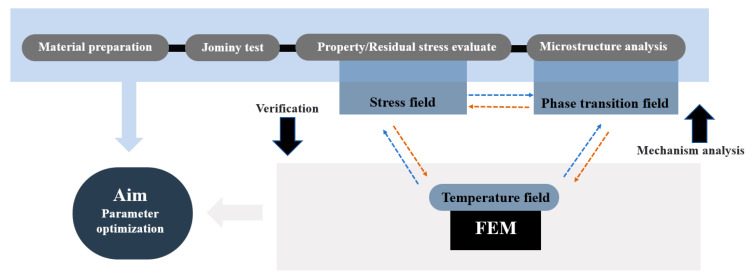
Research flowchart.

Addressing the requirements of the microstructure and property regulation of 9Cr-3W-3Co material during heat treatment, end hardening tests, residual stress measurements, and microstructural characterization were carried out in this study, aiming to clarify the microstructure and stress evolution law and explore the optimal heat treatment process parameters. The remainder of this paper is divided into three additional sections. The concrete implementation methods of heat treatment experiments, numerical simulation, and material characterization are introduced in Section 2. Focusing on the results and discussion, Section 3 comparatively examines the influence mechanisms of the austenitizing temperature on the hardness and residual stress distribution. The underlying mechanisms are discussed through microstructure/property/stress interrelationships, and corresponding optimization strategies for heat treatment processes are proposed. Finally, Section 4 summarizes the principal conclusions derived from this investigation. The research flowchart is shown in Figure 1.

## 2. Experimental Conditions and Approach on Simulation

### 2.1. Materials Composition and Experimental Methods

Samples were taken from adjacent locations on the same forging, where the as-forged microstructure was selected to investigate the hardenability, residual stress evolution, and microstructural changes under different austenitizing temperatures. This batch of materials was smelted and provided by the manufacturer. As presented in Table 1 and Table 2, the chemical composition of the 9Cr-3W-3Co martensitic heat-resistant steel used in this study was tested by chemical titration and EDS based on relevant national standards such as GB/T 223.4-2008 [15] and GB/T 5124.3-2017 [16]. The original microstructure exhibited an ASTM grain size number of 6.

**Figure 2:**
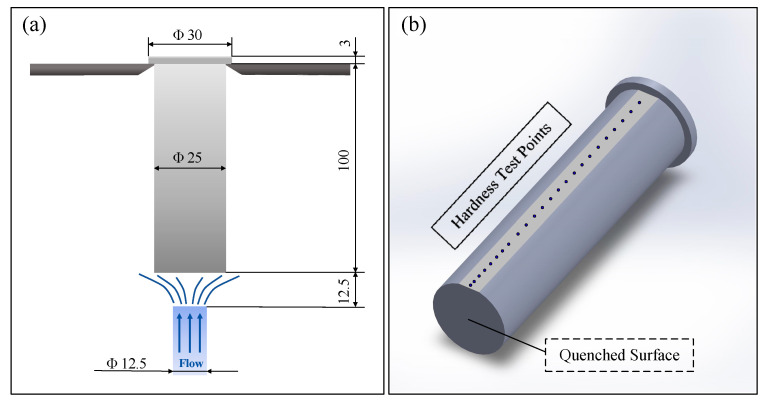
Schematic diagram of end-quenching test and sample detection: (**a**) end-quenching device and (**b**) hardness test sample.

**Figure 3:**
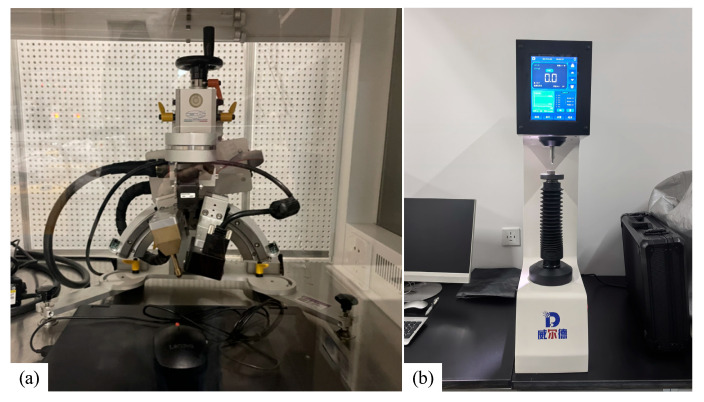
The test site: (**a**) platform of XRD to detect residual stress and (**b**) Rockwell hardness tester.

Based on the literature data [17], the density of the forged 9Cr-3W-3Co material is 7.88 g/cm^3^, with critical transformation temperatures determined as A_c1_ = 790 °C, A_c3_ = 865 °C, M_s_ = 345 °C, and M_f_ = 210 °C. Accordingly, the austenitizing temperature of the sample as a variable was set to 800 °C, 850 °C, 900 °C, 950 °C, and 1000 °C for this study. Following the standard GB/T 225-2006 [18] “Steel-Hardenability test by end quenching (Jominy test)”, cylindrical specimens (Φ 25 mm × 100 mm, as illustrated in Figure 2a) were heated to the specified austenitizing temperatures and held for 30 min, followed by end-quenching using a water spray apparatus. The free water column height was maintained at 65 ± 5 mm, with a 12.5 mm distance between the specimen end and spray nozzle. The local cooling rate progressively decreased with increasing distance from the quenched end. After the sample was cooled, 0.4 to 0.5 mm of thickness was ground off along the axial direction (as shown in Figure 2b), and the hardness was measured at certain intervals from the end, and the hardenability curve (hardness—distance curve) was drawn. The model of the Rockwell hardness tester used is WD-150RCA. The test site is shown in Figure 3a. A diamond indenter with an included angle of 120° was used. The pressure was 150 kgf and the holding time was 3 s.

X-ray diffraction (XRD), a non-destructive testing method integrating elasticity theory and diffraction principles, was employed to evaluate the surface residual stresses after quenching at varying distances from the quenched surface. The Spider X-EDGE portable X-ray diffractometer produced by the Italian GNR Company was selected as the detection equipment, as shown in Figure 3b. In order to fully study the effects of austenitizing temperatures on the residual stress, four control groups of 800 °C, 900 °C, 950 °C, and 1000 °C were set up, and the residual stresses were detected after quenching. Similar to the hardness test points, the test points of the residual stress were selected at 7 points close to the quenched surface, with 2 mm intervals from the end face up to 12 mm. Each measurement point underwent XRD scanning from −40° to 40°, with a 10° step interval. Finally, the microstructural evolution at different austenitizing temperatures was characterized by metallographic microscopy. After polishing, core samples were extracted from the quenched surfaces of specimens and etched using an FeCl_3_/HCl solution (20 mL HCl + 100 mL H_2_O + 5 g FeCl_3_) for 5 s.

### 2.2. Boundary Conditions of Simulation

**Figure 4:**
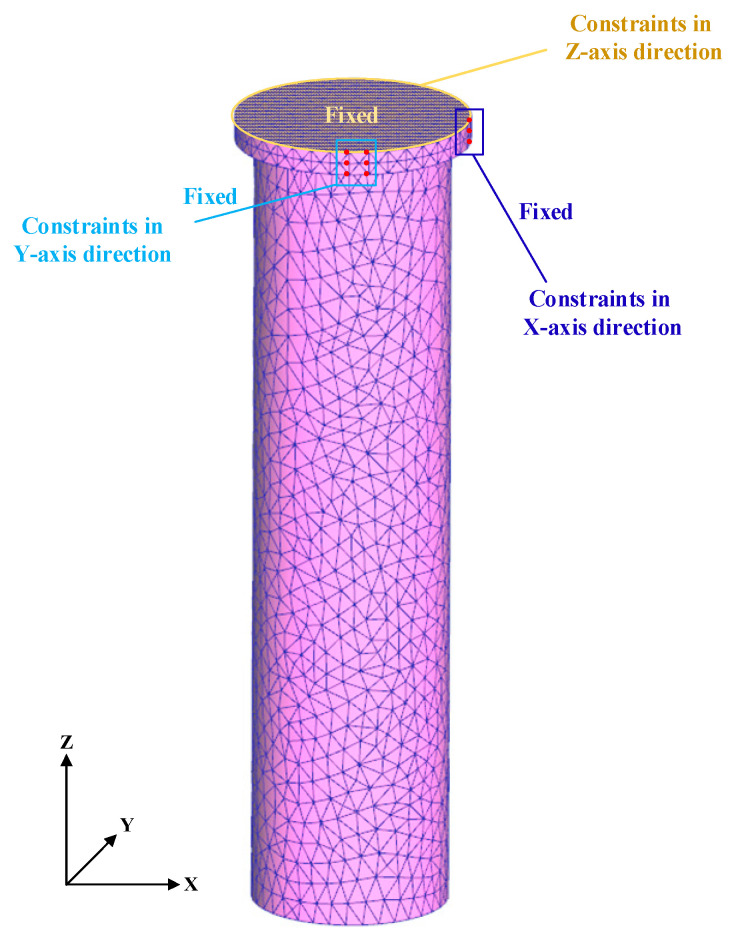
The mesh configuration and applied boundary conditions.

To intuitively reflect the influence of temperatures and stress fields on post-quenching microstructures and explore their internal relationship during the end-quenching process, numerical simulations were performed using Deform-HT. Due to limited thermodynamic parameter studies for this advanced material, the alloy composition listed in Table 1 was input into JMatPro to calculate the thermal conductivity, thermal expansion coefficient, specific heat capacity, phase equilibria, and transformation parameters. These derived parameters were then imported into the Deform heat treatment module for end-quenching simulations. The entire component was discretized into a tetrahedral mesh comprising 16,814 elements using an adaptive meshing technique. The object type was defined as an elasto-plastic type. Figure 4 illustrates the mesh configuration and applied boundary conditions. For the step increment control, the time step size was dynamically adjusted based on the cooling rate and temperature, with the maximum step size constrained by a temperature criterion limiting the change to ≤5 °C per increment.

To model the temperature distribution evolution during the cooling process from three austenitizing temperatures, i.e., 800 °C, 900 °C, and 1000 °C, the Newton/Raphson iterative method and Sparse solver were adopted to simulate the strain distribution, while for the calculation of the temperature phase, the conjugate gradient solver was used to improve convergence and stability. The air temperatures were uniformly set at 20 °C, while other process parameters and boundary conditions remained unchanged.

Heat transfer during end-quenching involves intense convective heat exchanges, with latent heat from phase transformations incorporated into the temperature field analysis. The transient heat conduction process was governed by the unsteady Fourier thermal conduction differential equation in cylindrical coordinates [19]:

(1) $$\lambda \left(\frac{\partial^{2}T}{\partial r^{2}}\;+\;\frac{1}{r}\frac{\partial T}{\partial r}\;+\;\frac{\partial^{2}T}{\partial z^{2}}\right)\;+\;Q=\rho C_{p}\frac{\partial T}{\partial t}$$

where λ represents the thermal conductivity coefficient of the material (W·m^−1^·°C^−1^), T is the temperature (°C), t is the time (s), Q is the latent heat generated during phase transformations (W·m^−3^), ρ is the density (kg·m^−3^), and Cp is the specific heat capacity at a constant pressure (J·kg^−1^·°C^1^).

**Figure 5:**
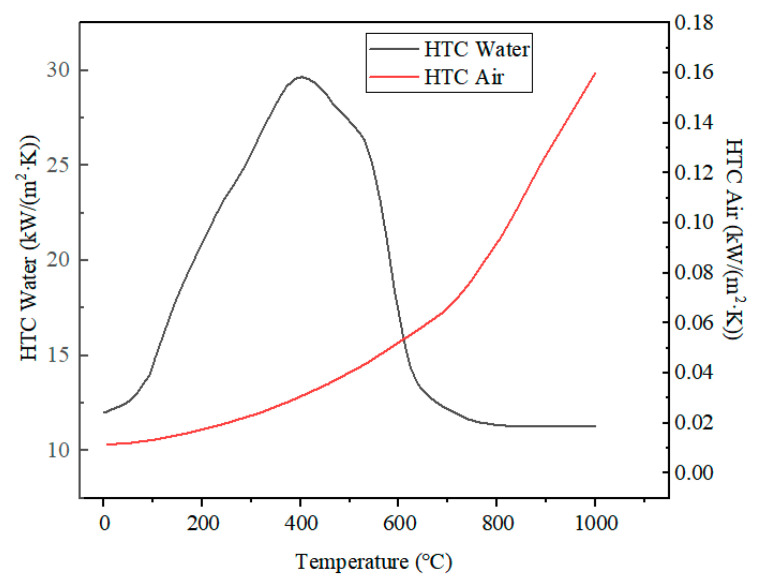
Heat transfer coefficient (HTC) in Jominy test.

During the end-quenching process, convective heat transfer occurs between the specimen quenching surface and cooling water, while the side and top surfaces experience combined convective and radiative heat transfer with the ambient air. The heat transfer coefficient consists of both convection and radiation components, with convection playing the dominant role. The convective heat transfer coefficients for the quenching medium and the radiative coefficient were selected based on [20]. The availability of the model was demonstrated by sensitivity analysis within a variation of values in the Jominy test. The HTC is shown in Figure 5, and the radiative coefficient was set to 0.8.

Since the entire phase transformation process is dominated by the austenite/martensite phase transformation process, which is a non-diffusive phase transformation, the Koistinen/Marburger model [21] was adopted to describe the martensite phase transformation process:

(2) $$V=1-\exp\left[-\partial \left(M_{s}-T\right)\right]$$

where V signifies the volume fraction of martensitic transformation, M_s_ is the starting temperature of the martensitic transformation, T is the quenching temperature, and ∂ is the kinetic constant of the phase transformation, obtainable from the TTT diagram.

## 3. Results Discussion

To clarify the influence of the temperature variation process at distinct locations on the microstructural evolution and residual stresses of the specimen, thermodynamic calculations and temperature field simulation results are preliminarily summarized. Subsequently, the underlying mechanisms governing the entire process are systematically investigated through integration with experimental findings.

### 3.1. Thermodynamics Calculations

**Figure 6:**
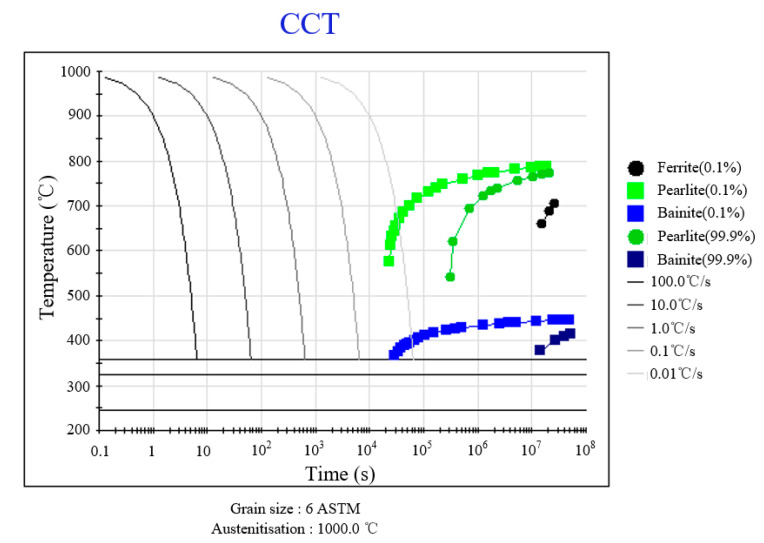
The resultant continuous cooling transformation (CCT) diagram.

Thermodynamic simulations of phase transformation behavior during continuous cooling were conducted using JMatPro 7.0 software, with the resultant continuous cooling transformation (CCT) diagram presented in Figure 6. As shown in the diagram, a complete martensitic microstructure can be achieved when the cooling rate exceeds 0.3 °C/s, indicating the theoretically excellent hardenability of the 9Cr-3Co-3Mo material. During the austenite-to-pearlite/bainite transformation, alloying elements with high concentrations (Cr, Mn, Mo, W) undergo a diffusion-driven redistribution. However, their diffusion rates are significantly slower than that of carbon [22], consequently shifting the bainite transformation curve in the CCT diagram towards the right. This phenomenon enables the material to obtain a desirable quenched martensitic microstructure under air-cooling conditions. According to the calculation result, when cooling from 1000 °C, the starting temperature (M_s_) and end temperature (M_f_) of the martensitic transformation were 353 °C and 224.5 °C, respectively, which are closely aligned with previously reported values in the literature.

### 3.2. Heat Transfer and Temperature Field by FEM

The distribution and evolution of the temperature field during the end-quenching process were simulated by the finite element method to analyze the mechanistic effects of temperature on microstructure transformation and stress distribution.

#### 3.2.1. Overall Axial Temperature Gradient

**Figure 7:**
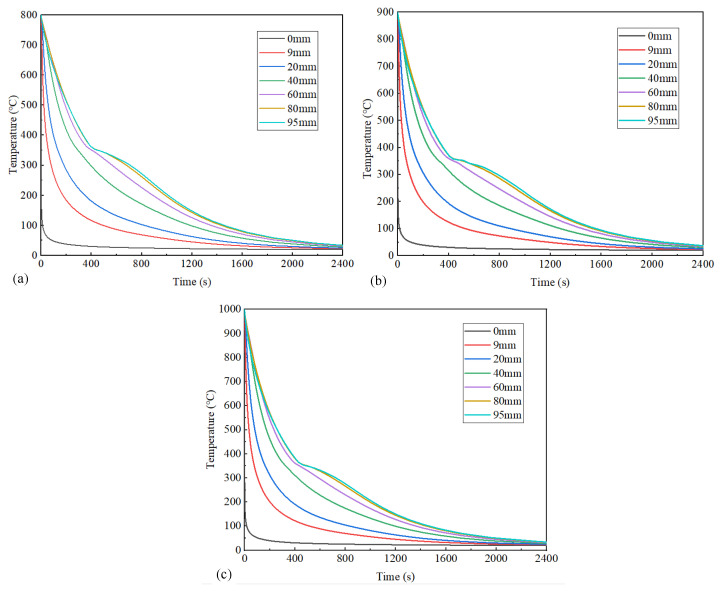
Surface cooling curves at different distances from quenched surface at austenitizing temperatures: (**a**) 800 °C, (**b**) 900 °C, and (**c**) 1000 °C.

In order to determine the overall cooling state of the specimens at different austenitizing temperatures, Figure 7 illustrates the surface temperature profiles at varying distances from the quenched surface under three distinct initial austenitizing temperatures. After the 2400 s cooling process, the temperature of each point gradually drops from an austenitizing temperature to room temperature. The statistical analysis of cooling rates revealed that temperatures near the end-quenched surface rapidly dropped below 210 °C (i.e., M_f_) within an extremely short duration. Notably, cooling rates progressively decreased with increasing distance from the quenched surface, demonstrating that the intrinsic limitation of hardenability was governed by specimen thickness.

Cross-referencing the cooling curves with the CCT diagram in Figure 6 confirms the absence of non-martensitic transformations during cooling. Consequently, the monotonic cooling path ensures an exclusive martensitic microstructure formation across the entire specimen under the investigated quenching conditions.

**Figure 8:**
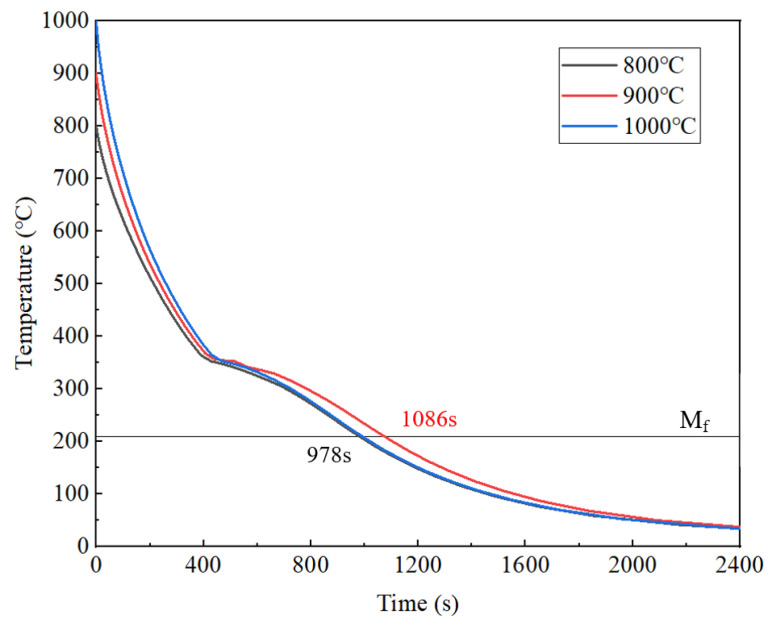
Cooling curve at a distance of 95 mm from the quenched surface.

To further demonstrate the influence of the austenitizing temperature on martensitic transformation, the cooling curve at 95 mm from the quenched surface is shown in Figure 8. The figure analysis reveals distinctive inflection points in cooling curves near the starting temperature of the martensite transformation (M_s_ = 353 °C), accompanied by abrupt cooling rate reductions that intensify with an increasing distance from the quenched surface. During the phase transformation from austenite (γ-Fe) to martensite (α′-Fe), the sudden release of latent heat induced localized thermal gradients. In positions relatively close to the quenched surface (≤40 mm distance), the high cooling rates promoted the rapid dissipation of phase transformation enthalpy. When the distance exceeded 40 mm, however, attenuated thermal conduction allowed for the progressive dominance of transformation-induced latent heat accumulation. As exemplified by the cooling curve at a 95 mm distance, notable temporal discrepancies emerged when the surface temperature of the specimens dropped to 210 °C (M_f_) at three austenitizing temperatures. Specifically, specimens subjected to 800 °C and 1000 °C austenitizing temperatures achieved this critical phase transformation within about 978 s, whereas those processed at 900 °C exhibited a prolonged duration of about 1086 s. In the temperature range of the martensitic transformation (M_s_~M_f_), the average cooling rate was relatively low when the austenitizing temperature was 900 °C.

#### 3.2.2. Local Axial Temperature Gradient near the Quenched Surface

To study the mechanism of residual stress generation at the end and the effect of the temperature gradient on it, the temperature field near the quenched surface was evaluated separately.

**Figure 9:**
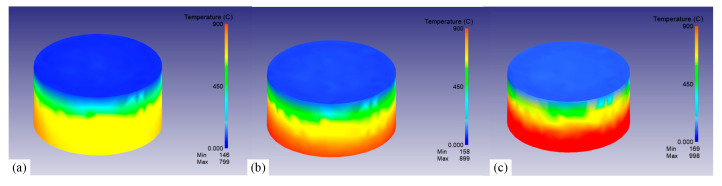
The temperature field within 12 mm near the quenched surface at 2.89 s under different austenitizing temperatures: (**a**) 800 °C, (**b**) 900 °C, and (**c**) 1000 °C.

**Figure 10:**
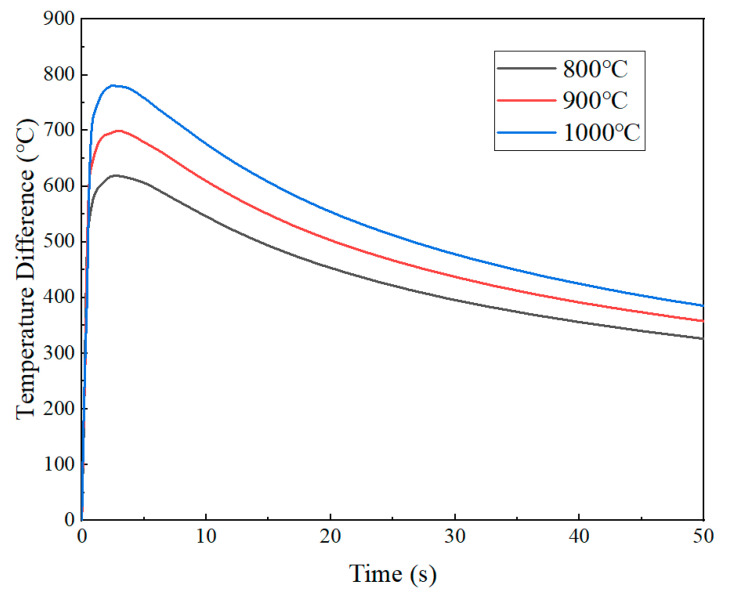
Temperature differential evolution at different austenitizing temperatures.

Figure 9 shows the temperature field within 12 mm from the quenched surface at 2.89 s under three austenitizing temperatures. It can be seen intuitively that the cooling rate gradually slowed down as the distance increased, inducing significant thermal gradients along the axial direction. As the austenitizing temperature increases, the temperature gradient tended to expand. Notably, the magnitude of these gradients demonstrates a positive correlation with the austenitizing temperature. Quantitative evaluations of maximum temperature differentials within the 12 mm region from the quenched surface during the initial 50 s cooling phase were conducted (Figure 10). During the initial quenching stage, instantaneous vaporization occurred as the quenched surface contacted the cooling medium, resulting in an abrupt escalation of local temperature differentials. As the austenitizing temperature increased from 800 °C to 1000 °C, the local temperature differentials caused by quenching gradually increased from 618.6 °C to 781 °C.

### 3.3. Effect of Austenitizing Temperature on Hardness and Microstructure

**Figure 11:**
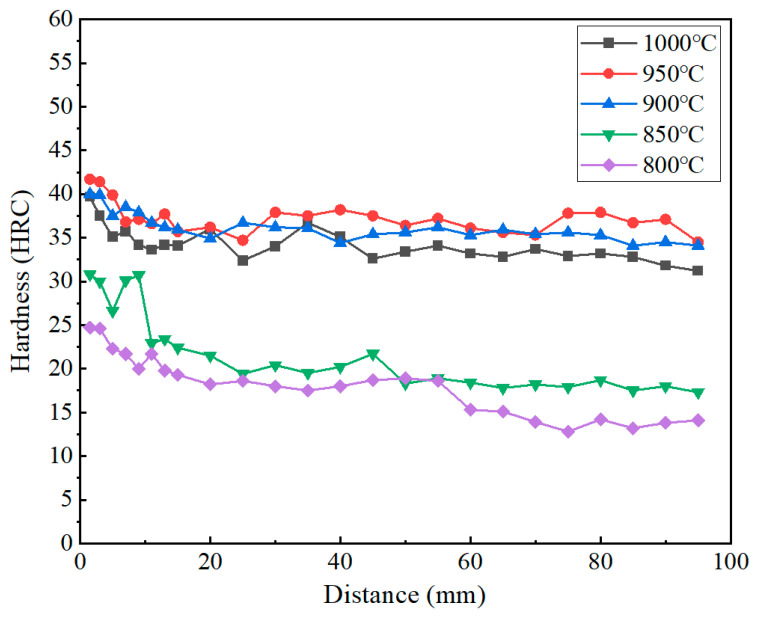
End-quenching hardness profiles at different austenitizing temperatures.

The hardness of martensitic microstructures after quenching can be influenced by multiple factors, including the cooling rate, prior austenite grain size, martensite lath dimensions, and the quantity/distribution of precipitates. End-quenching hardness profiles were systematically characterized for specimens at different austenitizing temperatures, with the results presented in Figure 11.

With the maximum hardness consistently appearing at the region near the quenched surface, all hardness curves exhibited similar trends. As shown in Figure 7, the hardness gradually decreased with an increasing distance from the quenched surface, corresponding to significant reductions in the cooling rate. Under slower cooling conditions, partial carbide precipitation would occur, leading to decreased carbon supersaturation in martensite and an increased martensite lath width. The above factors can contribute to the hardness reduction. The hardness profiles exhibited distinct variations under different austenitizing temperatures. The hardness of the specimens quenched at 900 °C and 950 °C decreased to a lesser extent, maintaining approximately 35 HRC within the distance range of 5~95 mm. And at the austenitizing temperature of 1000 °C, the hardness showed a slight decrease overall, but it remains in the range of 31 HRC to 35 HRC, indicating the excellent hardenability of the material. In contrast, specimens quenched at 800 °C and 850 °C displayed significant hardness gradients with substantially lower overall hardness values compared to other control groups. A sharp hardness decrease to below 20 HRC was observed within just 10 mm from the quenched end. These results clearly demonstrated the substantial influence of the austenitizing temperature on the post-quench hardness, with the following descending order of hardness values: 950 °C > 900 °C > 1000 °C > 850 °C > 800 °C. The subsequent microstructural analysis will provide further insights.

**Figure 12:**
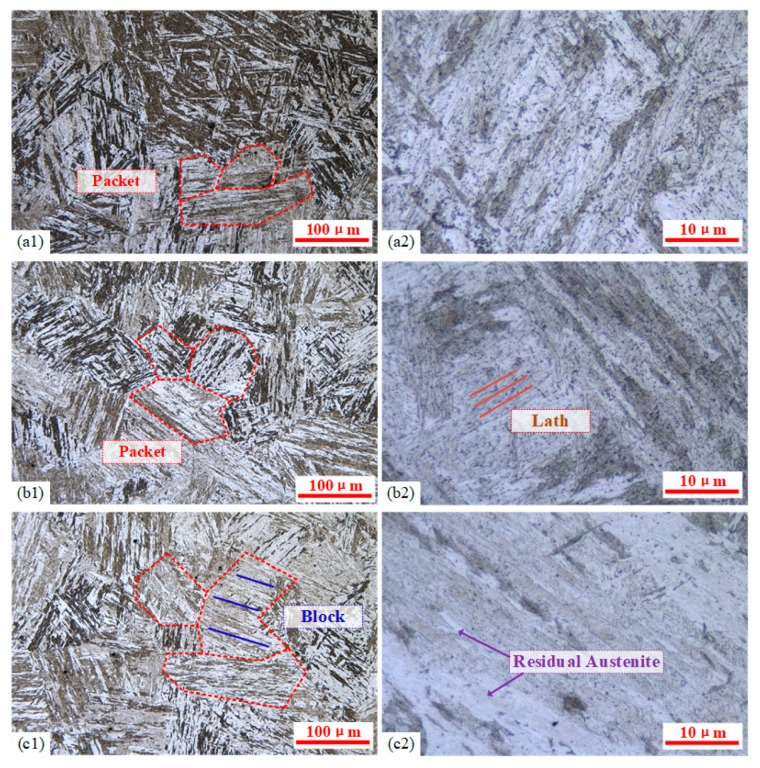
The martensitic microstructure formed at different austenitizing temperatures on the quenched surface: (**a1**,**a2**) 900 °C, (**b1**,**b2**) 950 °C, and (**c1**,**c2**) 1000 °C.

The lath martensite structure consists of packets, with each packet further divided into several parallel black-and-white regions known as blocks, which are composed of numerous parallel-aligned laths. As shown in Figure 12 at 100× and 1000× magnifications, all three austenitizing temperatures produced relatively uniform, typical lath martensite microstructures. Material hardness is determined by multiple factors, including a solid solution content of alloying elements, grain size, and retained austenite. Higher austenitizing temperatures can accelerate the dissolution of carbides and nitrides into austenite, promoting compositional homogenization. This phenomenon increases the content of carbon and alloying elements in martensite after quenching, thereby enhancing hardness through solid solution strengthening. Therefore, appropriate increases in the austenitizing temperature can improve post-quench mechanical properties.

The precipitates of 9Cr-3W-3Co after quenching are mainly M_23_C_6_ and MX, and there will be a precipitated Laves phase after a long time of aging [23]. At 1000× magnification (Figure 12(a2,b2,c2)), black particulate precipitates (carbides formed by elements such as Cr, W, Co, and Mn) were observed along prior austenite grain boundaries and martensite lath boundaries. Experimental results showed that as the austenitizing temperature increased, precipitates decreased significantly, enhancing solid solution strengthening after quenching. This microstructure difference can explain the higher hardness at 950 °C compared to 900 °C. However, when the austenitizing temperature reached 1000 °C, nearly all carbides and nitrides dissolved, and the hardness decreased slightly instead. This phenomenon is related to two other factors.

On the one hand, according to the measurement results, with the increasing austenitizing temperature, although the width of martensite laths remains nearly unchanged within a range of 0.4~0.6 μm, the sizes of both packets and blocks exhibit a significant increase accompanying the grain growth. Due to the excessively high temperature, the severe coarsening of both grains and martensite structures was observed at the austenitizing temperature of 1000 °C (Figure 12(c1)). On the other hand, elevated austenitizing temperatures promoted the dissolution of carbides such as Fe3C and Cr23C6, while simultaneously depressing the M_s_ point, resulting in an incomplete martensitic transformation at room temperature, and consequently, an increased retained austenite content (Figure 12(c2)). Therefore, excessively high austenitizing temperatures can lead to hardness reduction. The above two softening mechanisms were particularly pronounced at the austenitizing temperature of 1000 °C. Although the packet width in Figure 12(b1) showed a relatively minor increase compared to Figure 12(a1), the retained austenite content demonstrates no significant variation (Figure 12(a2,b2)). Consequently, under the 1000 °C austenitizing temperature, the combining effects of the reduced Hall/Petch strengthening and increased retained austenite content dominate over the effect of the solid solution strengthening contribution, ultimately resulting in an observable decrease in material hardness.

**Figure 13:**
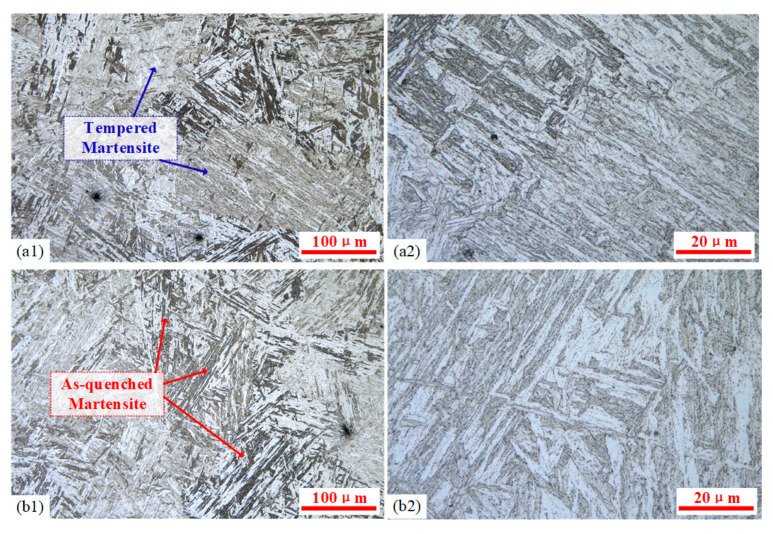
The martensitic microstructure formed at different austenitizing temperatures on the quenched surface: (**a1**,**a2**) 800 °C and (**b1**,**b2**) 850 °C.

When the quenching process was performed at temperatures above A_c1_ and below A_c3_, the microstructural transformation underwent a selective phase transformation. The regions with higher energy such as grain boundaries, dislocations, and martensitic lath boundaries in the original structure were preferentially nucleated and partially austenitized. The microstructures obtained by quenching at austenitizing temperatures of 800 °C and 850 °C are shown in Figure 13. The microstructure obtained at temperatures just above A_c1_ demonstrated characteristic features of incomplete austenitization. The formation of stable chromium carbides significantly reduced the diffusion capacity of carbon, thereby impeding the austenitization process. This resulted in a dual-phase microstructure consisting primarily of tempered martensite with limited as-quenched martensite. Compared with fully austenitized specimens, the constrained phase transformation leaded to an overall reduction in hardness. The comparison of the martensite lath morphologies in Figure 13(a1,b2) shows that during holding at 850 °C, coalescence occurred between adjacent laths in the lath martensite microstructure. And the tempered martensite laths formed by quenching were significantly thicker, with the thickness of the martensitic slats reaching 1.2~2.0 μm. Furthermore, the austenitization process progressed further during the insulation process. Consequently, the proportion of as-quenched martensite in the microstructure increased and the hardness was improved.

Considering the microstructure and hardness, quenching at 950 °C can achieve a better balance between the size of austenite grains, martensite lath dimensions, as well as the influence of the degree of the alloy element solid solution on hardness, and achieve the effective improvement of mechanical properties on the basis of ensuring good hardenability.

### 3.4. Residual Stress Analysis

#### 3.4.1. Formation Mechanism of Residual Stress in Quenching Process

The evolution of stress fields in materials results from the coupled effects of thermal and phase transformation behaviors. For 9Cr-3W-3Co steel, the residual stress primarily originates from the superposition of non-uniform cooling rates and martensitic transformation between surface and core regions [14]. The analysis is conducted in stages from the above two aspects.

During the cooling process of the specimens, due to the relatively slow cooling rate at the deeper regions, which are far from the surface, this temperature gradient will lead to differences in the degree of the volume contraction of the material in the depth direction. In the initial stage of cooling, both the surface and the core have relatively high temperatures, and the core material undergoes plastic deformation along with the outer layer material. The flow stress of the material under high temperatures remains relatively low; thus, the resulting thermal stress is also negligible. Furthermore, as the temperature of the near-surface materials decreases below the critical plastic flow temperature, the material in these regions gradually transition to the elastic state. The cooling causes a further volume contraction in these regions. the high-temperature yield strength of the internal material is relatively low, and the internal stress caused by the volume inhomogeneity can be released to some extent through the plastic compression of the core material and the tensile of the surface material. Nevertheless, the stress in this stage still leaves the surface material in a temporary state of tensile stress. Finally, while the surface material has largely completed its cooling and contraction, the inner layer begins to cool rapidly. The volume contraction of the inner layer material is restrained by the surface material, which has already been in the elastic state, leading to significant compressive stress at the surface and tensile stress within the core. Consequently, when solely considering the thermal effect, the surface material experiences a transition from tensile to compressive stress.

In addition to the thermal effects described above, high-alloy martensitic steels typically exhibit remarkable volume expansion during phase transformation, reaching 2~4%. The transformation stress is often the dominant factor in residual stress generation. The stress induced by the phase transformation will be superimposed with thermal stress. At the beginning of cooling, the surface layer undergoes a martensitic transformation first, and the volume expansion is constrained by the austenitic core material, generating a high surface compressive stress. Subsequently, as the core cools, its expansion is restricted by the surface layer. The inversion of the state of the surface compressive stress caused by the phase transition will reduce the surface compressive stress and even convert it to the surface tensile stress. Due to the delayed phase transformation, the transformation-induced stress may superimpose with or counteract the surface tensile stress occurring during the intermediate stage of thermal effects. The instantaneous maximum stress may even exceed the yield strength of the material. Failure to adequately control these quenching-induced internal stresses may result in severe material failures.

#### 3.4.2. Effect of Austenitizing Temperature on Residual Stress

**Figure 14:**
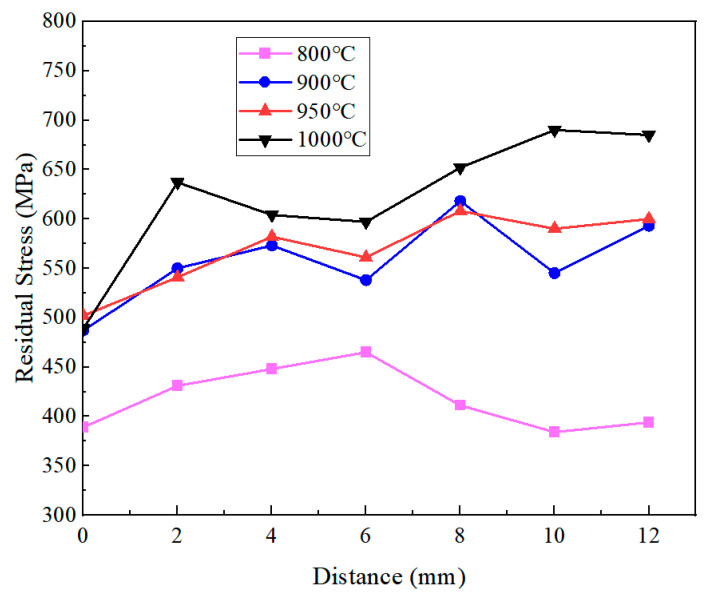
Axial residual stress at different austenitizing temperatures.

The residual stresses within a 12 mm distance from the quenched surface detected by X-ray diffraction were negative, indicating that the outer surface after quenching exhibited compressive residual stresses. The distribution of residual stresses along the axial direction is presented in Figure 14 after converting all stress values to their absolute magnitudes. The residual compressive stress gradually increased as the austenitizing temperature rose from 800 °C to 1000 °C. At 800 °C, the relatively small proportion of martensitic transformations, combined with the reduced flow stress of tempered martensite (due to carbide precipitation and decreased dislocation density), facilitated stress relaxation during quenching, resulting in the lowest residual stress. In contrast, the specimens austenitized at 1000 °C exhibited the highest residual stress after quenching, reaching a maximum value of 690 MPa at a 10 mm distance from the quenched surface. According to the temperature field simulation results shown in Figure 10, during the initial cooling stage, the specimen exhibited a significant axial temperature gradient. The material near the quenched surface transformed into martensite with a high yield strength, while the regions farther away remained untransformed. The subsequent cooling and contraction of the untransformed regions was constrained by the transformed martensite, generating substantial compressive stress on the surface. As the austenitizing temperature increased, the temperature difference within the 12 mm region gradually increased, thereby exacerbating the accumulation of thermal stress.

When the austenitizing temperature was set at 900 °C and 950 °C, the residual stresses formed at equivalent distances was 100 MPa less than that at 1000 °C. The reasons can be analyzed based on the cooling curves and microstructural characterization results shown in Figure 8. On the one hand, the microstructure after quenching at 900 °C exhibited more complete martensitic transformation and contained less retained austenite, thereby releasing a greater latent heat of the phase transformation. Similar to the constraint effect of the surface material on deeper regions, during the later stage of transformation stress evolution, the material near the quenched surface had already completed the martensitic transformation, while the expansion of the portion farther from the quenched surface was constrained. This transformation stress tended to generate residual tensile stress in the already-transformed regions, thereby partially offsetting the compressive stress caused by thermal effects. On the other hand, smaller quenching temperature differences helped reduce thermal stress, while greater transformation latent heat contributed to a localized cooling rate reduction (Figure 8), thereby decreasing the thermal stress caused by the temperature gradient. Under the combined effects of these factors, the maximum residual compressive stress formed during quenching at 950 °C decreased to 608 MPa. Compared with the 900 °C treatment, although the 50 °C temperature increase would elevate the thermal stress to some extent, the moderate rise in austenitizing temperature facilitated the enhanced dissolution of carbon and alloying elements. The increased solid solution content resulted in higher transformation latent heat during the quenching process, which effectively prevented the excessive accumulation of residual stresses. In summary, the austenitizing temperature of 950 °C was demonstrated to be the most favorable condition for achieving reduced residual stress levels.

## 4. Conclusions

Through a coupled numerical simulation and Jominy test integrating the temperature field, phase change field, and stress field analyses, the microstructure evolution, mechanical properties, and residual stress distribution of 9Cr-3W-3Co martensitic heat-resistant steel under different austenitizing temperatures (800–1000 °C) were investigated in this study. The principal findings are as follows:

(a) The austenitizing temperature significantly influences hardenability and hardness. Quenched at 950 °C, the material developed an optimal microstructure featuring moderately sized martensite lath packets and a relatively complete carbide dissolution, which ensures a remarkable solid solution strengthening effect. This austenitizing temperature can simultaneously prevent the hardness degradation associated with grain coarsening and excessive retained austenite formation, thereby maintaining a hardness above 35 HRC within a distance of 50 mm from the quenched surface.

(b) The quenching residual stresses are predominantly determined by the coupled effects of thermal stress and phase transformation stress. Increasing the austenitizing temperature (e.g., to 1000 °C) led to greater axial temperature gradients, resulting in significantly enhanced residual compressive stresses (peaking at 690 MPa). In contrast, with an austenitizing temperature of 950 °C, the release of transformation latent heat combined with moderate temperature differences effectively reduced residual stresses to 608 MPa, achieving an optimal balance between microstructural properties and stress control requirements.

(c) Based on the comprehensive evaluation of microstructural characteristics and residual stress distribution, 950 °C is identified as the optimum austenitizing temperature that achieves an ideal balance between high hardness with good hardenability and maintained residual stresses at relatively low levels. This study provides fundamental theoretical insights and practical optimization approaches for the thermal manufacturing processes of large-scale forgings.

## Figures and Tables

**Table 1:** Chemical composition of 9Cr-3W-3Co.

Element	C	Si	Mn	S	Cr	Ni
**Content (wt.%)**	0.081	0.16	0.49	0.0024	8.93	0.044
**Element**	V	Co	W	Nb	B	Al
**Content (wt.%)**	0.23	3.08	2.52	0.06	0.022	0.013

**Table 2:** Mechanical properties of 9Cr-3W-3Co at normal temperature.

Material	Rm/MPa Tensile Strength	Rp0.2/MPa Yield Strength	A/% Elongation	Z/% Rate of Reduction
9Cr3W3Co	1027	983	18.5	20.11

## Data Availability

The original contributions presented in this study are included in the article.

## References

[1] He X.K., Liu Z.D., Wang T.J., Peng J.Q., Nie Y.H., Shen G.Q. (2023). Development progress of heat-resistant materials and forgings for ultra-supercritical steam turbine rotor in China. China Metall..

[2] Wang D.X., Du J.F., Sun S.W., Yang H.S., Li L.P., Qiao Z. (2024). Development of 9–12% Cr Heat-resistant Steels Used for Ultra-Supercritical Steam Turbine Rotor. Dongfang Turbine.

[3] Yan W., Wang W., Shan Y.Y., Yang K. (2013). Microstructural stability of 9–12%Cr ferrite/martensite heat-resistant steels. Front. Mater. Sci..

[4] He H.S., Yu L.M., Liu C.X., Li H.J., Gao Q.Z., Liu Y.C. (2022). Research Progress of a Novel Martensitic Heat-Resistant Steel G115. Acta Metall. Sin..

[5] Yan P., Liu Z.D., Bao H.S., Weng Y.Q., Liu W. (2014). Effect of normalizing temperature on the strength of 9Cr-3W-3Co martensitic heat resistant steel. Mater. Sci. Eng. A.

[6] Yan P., Liu Z.D., Bao H.S., Weng Y.Q., Liu W. (2014). Effect of tempering temperature on the toughness of 9Cr-3W-3Co martensitic heat resistant steel. Mater. Des..

[7] Li Q., Chen Z.Z., Jiang X.L. (2021). Development and engineering manufacture technology of 9~12% Cr high and medium pressure rotor material. Iron Steel.

[8] Zhu H.B., Zhao Y.Q., Shi S., Huang X.G., Wang X.M. (2024). Analysis on austenite continuous cooling behavior and hardenability of 1300 MPa medium-thick plates. China Metall..

[9] Wang B.B., Zhu X.D., Zhang L.C., Zhou X.Y., Wu H.H., Wang S.Z., Wu G.L., Gao J.H., Zhao T.H., Mao X.P. (2024). Influence of typical elements and heat treatment parameters on hardenability in steel: A review. J. Iron Steel Res. Int..

[10] Jin M., Lian J.S., Jiang Z.H. (2006). A new method for prediction of Jominy curve of structural steel. Acta Metall. Sin..

[11] Zhu Y.F., Gu J.L., Zhou H.H., Wang Z.X., Zhang J.W. (2022). End-quenching experimental curves and simulation calculation of 18CiNiMo7-6 steel for high-speed railway gears. Heat Treat. Met..

[12] Lu S.M., Wan L., Zheng S.J., Li M.N., Xu Z.D., Wang H. (2024). Numerical simulation and experimental study of temperature field and microstructure field during quenching of nuclear steel. J. Cent. South Univ. Sci. Technol..

[13] Yang Z.Y., Wang M., Zhang M.M., Shao C., Wang H. (2025). Analysis of the Causes and Control of High Hardenability of Gear Steel 18CrNiMo7-6HL. JOM.

[14] Feng X.W., Wang Y.X., Han J.X., Li Z., Jiang L., Yang B. (2024). Numerical Simulation and Experimental Verification of the Quenching Process for Ti Microalloying H13 Steel Used to Shield Machine Cutter Rings. Metals.

[15] (2008). Alloyed Steel-Determination of Manganese Content-Potentiometric or Visual Titration Method.

[16] (2017). Hardmetals—Part 3: Determination of Cobalt-Potentiometric Method.

[17] Yang L.X., Ma L.T., Chen Z.Z., Li X.J. (2018). Effect of tempering temperature on microstructure and hardness of 9Cr-3W-3Co martensitic steel. Heat Treat. Met..

[18] (2006). Steel-Hardenability Test by End Quenching (Jominy Test).

[19] Feng X.W., Zhang K., Liu J.J., Shi R.X., Sun B.W., Liu S., Li Z.P., Yang B. (2024). Numerical simulation and experimental verification of heat treatment after forging of SA508-3 steel for steam generator. Heat Treat. Met..

[20] Medina-Juárez I., Oliveira D.A.J., Moat J.R., Garcia-Pastor F.A. (2019). On the Accuracy of Finite Element Models Predicting Residual Stresses in Quenched Stainless Steel. Metals.

[21] Koistinen D.P., Marburger R.E. (1959). A general equation prescribing the extent of the austenite-martensite transformation in pure iron-carbon alloys and plain carbon steels. Acta Metall..

[22] Landgraf P., Birnbaum P., Meza-García E., Grund T., Kräusel V., Lampke T. (2021). Jominy End Quench Test of Martensitic Stainless Steel X30Cr13. Metals.

[23] Li H.Z., Liang J., Xu L.Y., Wang B. (2017). Effect of cyclic normalization on microstructure and impact toughness of 9Cr3W3Co steel. Trans. Mater. Heat Treat..

